# A Pilot Study on Tocilizumab for Treating Refractory Adult-Onset Still’s Disease

**DOI:** 10.1038/s41598-017-13639-y

**Published:** 2017-10-18

**Authors:** Ting Li, Liyang Gu, Xiaodong Wang, Li Guo, Hui Shi, Chengde Yang, Sheng Chen

**Affiliations:** 1 Department of Rheumatology, Ren Ji Hospital, School of Medicine, Shanghai Jiao Tong University, Shanghai, 200001 China; 2 Department of Rheumatology, Ren Ji Hospital South Campus, School of Medicine, Shanghai Jiao Tong University, Shanghai, 201112 China; 3 Department of Rheumatology and Immunology, Ruijin Hospital, School of Medicine, Shanghai Jiao Tong University, Shanghai, 200025 China

## Abstract

To investigate the efficacy and safety of Tocilizumab (TCZ) in patients with refractory adult-onset Still’s disease (AOSD). We enrolled 8 female patients from October 2013 to July 2014. All patients fulfilled Japan’s Yamaguch AOSD classification and recognized as refractory AOSD. All Patients received TCZ treatment 4–8 mg/kg every 4 weeks. Evaluation of efficacy was conducted after 3 months and 6 months, including clinical manifestations of AOSD patients, improvement of inflammatory markers as well as glucocorticoids dosage adjustments. Treatment-related adverse events were also recorded. Patients treated with Tocilizumab with average age 41.1 years old, the average disease duration 23.6 months. Two patients drop off due to infusion side effects. Others were followed at least 6 months. After 3 months of follow-up, remission rates of fever, arthritis and rashes from 8 patients were 87.5%, 100% and 87.5%. White blood cell counts, erythrocyte sedimentation rate, C-reactive protein and ferritin levels were decreased (P < 0.01) significantly compared to treatment before. Furthermore, the average dose of prednisone was reduced from 51.7 ± 38.4 mg/d to 12.9 ± 7.7 mg/d (P < 0.01). Our findings suggest that tocilizumab could alleviate the clinical manifestations of refractory AOSD rapidly and efficiently.

## Introduction

Adult Still’s disease (adult-onset Still’s disease, AOSD) is a clinical syndrome of unknown etiology, with intermittent fever, rashes, arthritis or joint pain, sore throat as main clinical manifestations, accompanied by increasing number of peripheral blood leukocytes and granulocytes, hepatic dysfunction and involvement of multi-systems^1^. Studies have shown that the levels of inflammatory cytokines in the peripheral blood of AOSD patients are significantly increased, such as interleukin (IL) -1, IL-6, IL-18, tumor necrosis factor-α (TNF-α), interferon-γ (IFN-γ), suggesting that cytokines play an important role in the pathogenesis of this disease^2^. The treatments for AOSD include non-steroidal anti-inflammatory drug (NASID), glucocorticoids, and disease modifying anti-rheumatic drugs (DMARD). With understanding the pathogenesis of AOSD, biological treatments blocking pathogenic cytokines become new targets for the disease^3–7^.

It has been continuously reported in recent years that the IL-6 receptor monoclonal antibody (Tocilizumab, TCZ) therapy has favorable effect on recurrent AOSD or macrophage activation syndrome, (MAS)^6,7^, but most reports are based on small samples or case report. This study is aimed to evaluate the efficacy and safety of TCZ treatment for recurrent refractory AOSD.

## Result

### General information of patients

8 patients received TCZ were intravenously treated with TCZ every 4 weeks, including five cases at the dose of 8 mg/kg (maximum 400 mg), other three patients at the dose of 4 mg/kg. One patient got remission after received the first infusion of TCZ, however she experienced the allergic reactions (fever and rash) during the second infusion and stopped the TCZ treatment afterward. The remaining seven patients received at least 3 months of medication. In the 4th month, the treatment was terminated on another patient due to allergic reactions and relapse of AOSD. The remaining six patients received more than 6 months treatment and three of them dropped off the medication afterward. No relapses were observed during the follow-up duration with average 5 months (3–12 months). In addition, there are still three cases receiving TCZ therapy till now.

### Clinical efficacy and improvement of inflammatory markers after TCZ therapy

Before TCZ therapy, all of 8 patients had both fever and arthritis, 3 patients had rashes. After the initial infusion of TCZ, symptoms including fever and arthritis were all improved (8/8, 100%), rashes were improved in 76.7% of patients (2/3). After 3 months of follow-up, remission rates of fever, arthritis and rashes from 8 patients were 87.5% (7/8), 100% (8/8) and 87.5% (7/8); and remission rates were 75% (6/8), 100% (8/8) and 75% (6/8), after 6 months of follow-up, respectively. These indicated that TCZ could alleviate the clinical manifestations of refractory AOSD rapidly and efficiently.

**Figure 1:**
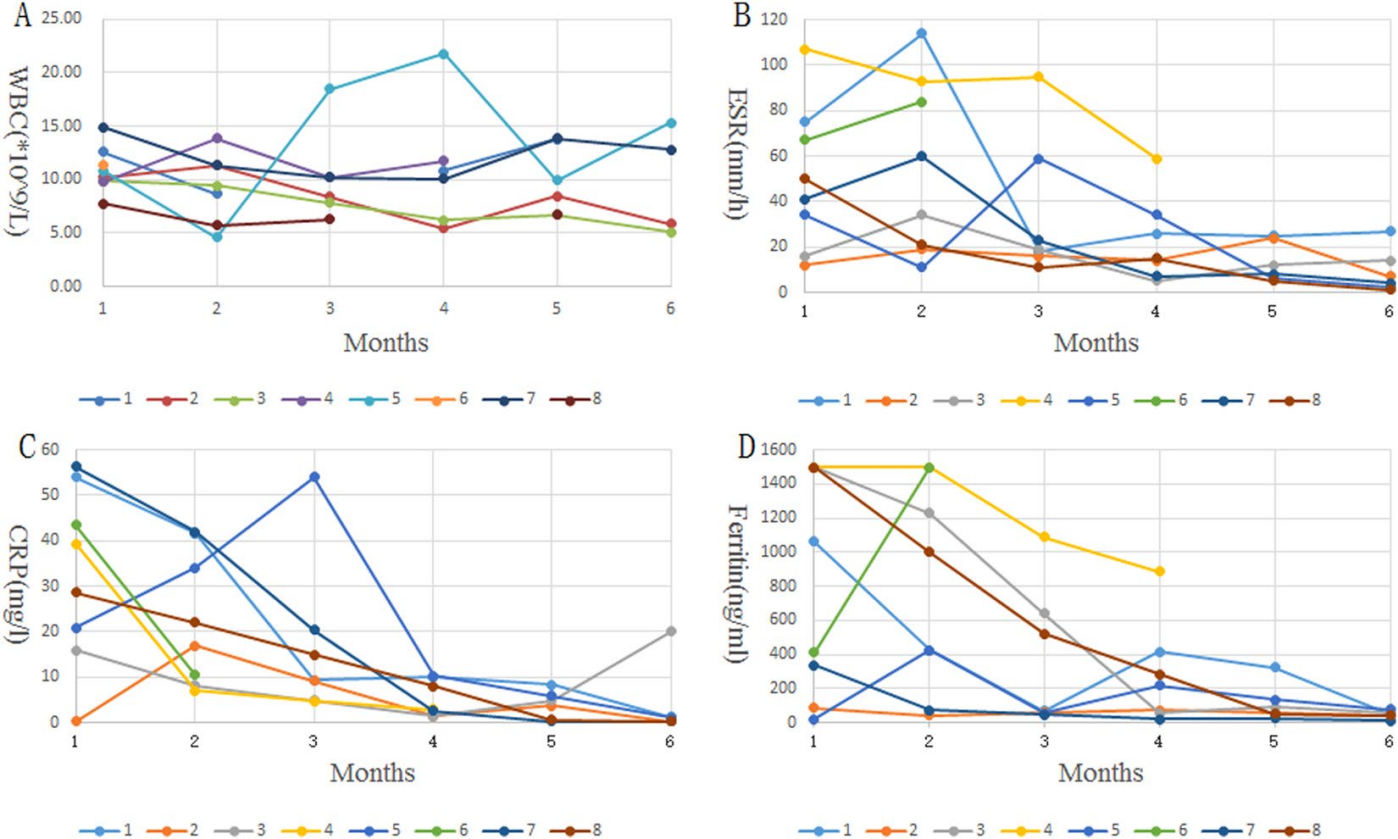
Clinical efficacy and improvement of inflammatory markers after TCZ therapy.

Before TCZ therapy, white blood cell count of 8 patients was 10.9*10^9^/L (7.78–14.9), ESR 50.3 mm/h (12–107), C-reactive protein 32.4 mg/L (0.2–56.4), ferritin level 801.4 ng/ml (18.6–1500), in average. After 3 months of treatment, white blood cell counts, erythrocyte sedimentation rate, C-reactive protein and ferritin levels were 10.3*10^9^/L (6.3–18.4), 29.3 mm/h (11–59), 16.5 mg/l (2.9–54), 324.9 ng/ml (48–886.4) and decreased (P < 0.01) significantly than before treatment. After 6 months, these indicators were 8.7*10^9^/L (5.1–15.3), 8.3 mm/h (1–27), 0.8 mg/l (0.2–1.5), 46.7 ng/ml (22.1–75) and, were significantly improved when compared with the ones before treatment and after 3 months. Among them, the most rapid decrease was observed in C-reactive protein, as shown in Fig. 1.

### Contribution of TCZ in reducing glucocorticoids in refractory AOSD treatment

**Figure 2:**
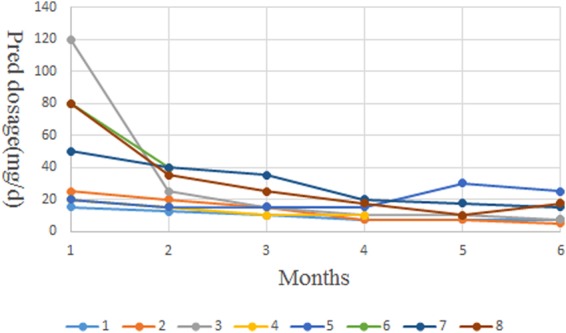
Contribution of TCZ in reducing glucocorticoids in refractory AOSD treatment.

Before treatment, 8 cases of AOSD patients received prednisone with an average dose of 51.3 mg/d (15–120). 3 months after treatment, the average dose of prednisone was 17.1 mg/d (10–35), with over 30% reduction in 86% patients. At 6 months after TCZ therapy, the average dose of prednisone was11.7 mg/d with further reduction compared with doses before and after 3 months of treatment. DMARD drugs were eventually withdrawn in 3 cases in this study. Two patients stop DMARDs at the beginning of treatment of TCZ (Cyclosporine A and Leflunomide) and one patient stop Leflunomide at the third month after treatment of TCZ. All these indicating the contribution of TCZ in rapidly reducing the glucocorticoids dosage during treatment of refractory AOSD, as shown in Fig. 2.

### Adverse events

During TCZ treatment, 2/8 patients (25%) had infusion related adverse reactions (which occurred in the first two months and four months, thus the TCZ treatments were terminated). During the follow-up, 3/8 patients (37.5%) had infections, including 2 cases of urinary tract infection and 1 case of upper respiratory tract infection. Coagulation abnormality and hepatic dysfunction deterioration occurred in one patient (12.5%) after TCZ therapy. The patient was diagnosed as MAS and the adverse effects were regarded as signs of deterioration. Her inflammatory markers were improved after intravenous gamma-globulin treatment, and there were no further adverse effects of her subsequent utilization of IL-6 receptor monoclonal antibody. Therefore, TCZ therapy is well tolerated in refractory AOSD patients in general.

## Discussion

AOSD is an autoimmune disease of unknown etiology. With complicated and diverse clinical manifestations, AOSD is more common in female, often with multi-system involvement. The ages of patients usually range from 16 to 35 years old^8^, while onsets at old ages have been reported as well^9^. Most AOSD patients have good responses to glucocorticoids. However, a small part of patients with severe clinical symptoms had poor responses to high dosage of glucocorticoids, or had difficulties in withdrawing the utilization of glucocorticoids even combined with immunosuppressive agents, further leading to the relapse of AOSD. These patients are defined as refractory AOSD patients, who need higher dosage of glucocorticoids, longer treatment duration and the simultaneous application of immunosuppressive drugs. Meanwhile, the treatment-related complications also increase with poor prognosis of refractory AOSD, which is crucial and difficult for treatment against AOSD^10,11^.

Recent studies have shown that monocytes - macrophage activation is central of the pathogenesis of AOSD. In active AOSD patients, activated monocytes macrophages can produce large amounts of cytokines such as TNF-α, IL-6, IL-1 and IL-18 and so on^12–15^. Wherein the IL-6 is associated with symptoms including fever, rash, leukocyte hyperplasia, elevated inflammatory markers and hepatic dysfunction in AOSD patients^2^. In recent years, many groups reported that they successfully treated AOSD with biologic therapies targeting at cytokines^16–20^. These biologic therapies are mainly for patients who had poor response to the combination of methotrexate or NSAIDs with glucocorticoids^3–7^.

This study aimed to evaluate the efficacy and safety of IL-6 receptor monoclonal antibody (Tocilizumab) therapy for Chinese patients with refractory AOSD. Eight patients with clinical refractory AOSD enrolled in our study, except one newly diagnosed patient, all the remaining cases had history of recurrences. The patients with AOSD recurrences were previously treated with high dosage of glucocorticoids (with no response or dependent on the dose) and several immunosuppressive therapies, including methotrexate, cyclosporine, or intravenous immunoglobulin therapy (with no response either). 1 patient have utilized infliximab with no effect. 5 patients (62.5%) had no response to over two different immunosuppressant or failed to tolerate them. After receiving TCZ at dose of 4–8 mg/kg/month for 3 months of follow-up, remission rates of fever, arthritis and rashes from 8 patients were 87.5% (7/8), 100% (8/8) and 87.5% (7/8); and remission rates were 75% (6/8), 100% (8/8) and 75% (6/8), after 6 months of follow-up, respectively. Inflammatory markers such as C-reactive protein, erythrocyte sedimentation rate, and ferritin were significantly decreased, and dosage of glucocorticoids can be rapidly and effectively reduced, all of which indicates the ideal effect of TCZ therapy for refractory AOSD patients. It is worth noting that the doses of TCZ at 4–8 mg/kg and the intervals ranging from 2–4 weeks are both referred from literatures published on TCZ therapy for AOSD patients, since there is no commonly accepted standard on dosage and dosing interval for TCZ therapy currently, and procedures in detail remains to be further investigated. We also observed that the decrease in CRP after TCZ therapy is particularly obvious. Some patients had CRP declined to normal level only on the next day even though they still had clinical symptoms at the time. The reason for the phenomenon may be the involvement of IL-6 in CRP production. Therefore, the decrease in CRP may not be a reliable indicator of clinical remission. At the 6th month of treatment, three of six patients with clinical remission ended the TCZ therapy, with no recurrence during followed-up till now. Three patients are still continuing with the TCZ therapy. Relapse of the disease only occurred to one case with attempts to reduce the TCZ dosage, following by remission again soon after the return to initial dosage of TCZ. Thus, TCZ therapy has satisfying effects on refractory AOSD patients in short-term.

TCZ therapy for AOSD patients complicated with MAS still remains controversial. It has been reported by Kobayashi *et al*. that MAS appeared after TCZ treatment, and improved by increasing dose of glucocorticoids and utilization of cyclosporine. In Kobayashi’s report, the subsequent utilization of TCZ therapy effectively prevented recurrence of symptoms during the follow-up of 171 days^21^. Here in our study, coagulation abnormalities and hepatic dysfunction deterioration occurred in one patient with newly onset refractory AOSD and MAS simultaneously (manifestations including hepatic dysfunction, decreased fibrinogen, MAS cells observed from bone marrow biopsy, no response to high doses of glucocorticoids). After TCZ treatment for 1 week, the symptoms of the AOSD patient were improved, however, the levels of liver enzymes and coagulation time of the patient were further deteriorated. Only after intravenous treatment of gamma-globulin for another week, the patient recovered. No recurrence or similar situation were found during subsequent follow-up. MAS, which indicates high disease activity and vulnerability of liver, is regarded as the severe complication of AOSD. Further studies are required to testify if combination of glucocorticoids and IL-6 receptor antagonist could be used to treat AOSD and MAS patients.

In this observational study, TCZ therapy is generally well tolerated in refractory AOSD patients. Only one case had adverse effect of hepatic dysfunction and coagulation function deterioration. Other adverse effects were infections (37.5%), infusion related reactions (25%). Adverse effects such as severe infections including tuberculosis, neutropenia, hyperlipidemia was not found in this study. There are still some limitations in our observational study including small samples and the limited follow-up. Further randomized and double, blind studies conducted from multi-centers are required to confirm the efficacy and safety of TCZ.

## Conclusion

The combination of TCZ with DMARDs or glucocorticoids can partially relieve symptoms of refractory AOSD patients, reduce inflammatory markers and contribute to withdrawal of glucocorticoids. Moreover, the therapy is generally well tolerated. Nevertheless, further prospective studies on this therapy are still required to evaluate the specific dosage and duration of the treatment. It is worth nothing that cautions of TCZ therapy are also required in AOSD patients, especially in patients with AOSD complicated with MAS.

## Subjects and Methods

Totally 8 female patients were enrolled from October 2013 to July 2014 at Department of Rheumatology, Ren Ji Hospital, Shanghai Jiao Tong University. These are hospitalized patients who were diagnosed as refractory AOSD, with average age 41.1 years old (21 to 63 years old), the average disease duration 23.6 months (3 to 84 months, defined from the start of disease onset). The most common symptoms were fever (8/8, 100%), joint pain (8/8, 100%), and rash (7/8, 87.5%), 6/8 patients had lymph nodes enlargement, 3 had liver and spleen enlargement, 2 had pleurisy. One case was newly diagnosed patients (complicated with MAS), while the others were recurrent AOSD. The patients with recurrent AOSD are previously treated with different doses of glucocorticoid and immunosuppressive therapy, including methotrexate, hydroxychloroquine, leflunomide, cyclophosphamide, mycophenolate mofetil, cyclosporine, thalidomide, triptolide and Intravenous immunoglobulin therapy. 1 patient received infliximab treatment with no effect. 5 patients (62.5%) failed to more than two different immunosuppressant drugs. All patients fulfilled 1992 Japan’s Yamaguch classification criteria for AOSD^1^, and were classified as clinical refractory AOSD. Refractory AOSD is defined as: 1. no response to high dosage of glucocorticoids (more than 2 mg/kg) or dependent on glucocorticoids, or 2. No response to immunosuppressant or intolerance on immunosuppressant previously; or 3. acute onset complicated with hepatic dysfunction or MAS. The characteristics of patients are shown in Table 1.

**Table 1:** General information of 8 refractory AOSD patients receiving TCZ therapy.

No. case		1	2	3	4	5	6	7	8
Gender		female	female	female	female	female	female	female	female
Age (years old)		21	47	63	62	32	34	24	46
Multi-system involvement		Y	Y	Y	Y	Y	Y	Y	
Disease duration (month)		12	6	3	14	84	20	24	26
Yamaguchi’s criteria (>5 criteria, at least 2 major criteria)									
Major Criterias	Fever ≥39.0 °C, over 1 week	Y	Y	Y	Y	Y	Y	Y	Y
	Arthritis over 2 weeks	Y	Y	Y	Y	Y	Y	Y	Y
	Typical rashes	Y	Y	Y	Y	Y		Y	Y
	White blood cells ≥10*10^9/L	Y	Y	Y		Y	Y	Y	
Minor Criterias	Sore throat	Y	Y		Y		Y		
	lymph nodes and (or) spleen enlargement			Y	Y	Y	Y	Y	Y
	Hepatic dysfunction	Y	Y	Y				Y	
	Negative RF and ANA	Y	Y	Y		Y	Y	Y	Y
Laboratory Examination	White blood cells (10^9/L)	12.9	13	17.7	9.78	16.4	14.9	11.3	7.78
	Ferritin (ng/mL)	1060.9	28.5	789.8	5135	18.6	334.8	412.3	1500
	ESR (mm/h)	75	6	55	93	34	41	76	50
	CRP (mg/l)	54	2.5	99.9	89.5	20.8	95	32.4	28
Previous Treatments	Pred maximum-current dosage (mg/d)	60–15	500–30	120–100	80–20	40–30	240–80	160–80	120–40
	Immunosuppressive drugs	MTX, HCQ, MMF, LEF	CsA, MTX, IVIG, Thalidu-mide	/	HCQ, T2, MTX	MTX CsA, LEF, IFX	MTX	MTX CsA	CTX
Dosage of TCZ	mg/kg,q4w	8 mg/kg, *13	4 mg/kg, *6	4 mg/kg *6	4 mg/kg, *4	8 mg/kg, *6	8 mg/kg, *2	8 mg/kg, *8	8 mg/kg, *7

MTX: methotrexate; HCQ: hydroxychloroquine; MMF: mycophenolate mofetil; LEF: leflunomide; CsA: cyclosporine A; IVIG: intravenous immunoglobulin; T2: tripterygium glycosides.

Patients received TCZ treatment after screening of relevant contraindications and signing the consent forms. They were intravenously treated with TCZ every 4 weeks, in which five cases at dose of 8 mg/kg (maximum 400 mg), and the others at a dose of 4 mg/kg (2 cases were older than 60 years old). The evaluation of TCZ treatment was conducted after 3 months and 6 months, including clinical manifestations of AOSD patients, improvement of inflammatory markers (such as erythrocyte sedimentation rate, C-reactive protein and ferritin) as well as glucocorticoids dosage adjustments. Treatment-related adverse events were also recorded.

This study was conducted in accordance with the principles expressed in the Declaration of Helsinki. The study was approved by the Institutional Review Board of Renji Hospital. The written informed consent were obtained from all 8 patients.
