# Publisher Correction: A Pilot Study on Tocilizumab for Treating Refractory Adult-Onset Still’s Disease

**DOI:** 10.1038/s41598-018-36662-z

**Published:** 2018-12-05

**Authors:** Ting Li, Liyang Gu, Xiaodong Wang, Li Guo, Hui Shi, Chengde Yang, Sheng Chen

**Affiliations:** 1 Department of Rheumatology, Ren Ji Hospital, School of Medicine, Shanghai Jiao Tong University, Shanghai, 200001 China; 2 Department of Rheumatology, Ren Ji Hospital South Campus, School of Medicine, Shanghai Jiao Tong University, Shanghai, 201112 China; 3 Department of Rheumatology and Immunology, Ruijin Hospital, School of Medicine, Shanghai Jiao Tong University, Shanghai, 200025 China

Correction to: *Scientific Reports* 10.1038/s41598-017-13639-y, published online 18 October 2017

This Article contained an error in the Acknowledgements section.

“This study was funded by Shanghai Municipal Commission of Health and Family Planning grant number 140613163312706.”

now reads:

“This study was funded by Shanghai Municipal Commission of Health and Family Planning grant number 201440040.”

In addition, the email addresses for Chengde Yang and Sheng Chen were incorrectly listed as 13917556052@139.com and yangchengde@hotmail.com respectively.

